# Dissenters and Medical Authority

**Published:** 1913-09-20

**Authors:** J. N. Beadles

**Affiliations:** The Hollies, Belle Vue Road, Cinderford, Gloucestershire


### EDITOR'S LETTER-BOX.

### Dissenters and Medical Authority.

*To the Editor of* The Hospital.

Sir,—In your interesting article in The Hospital of September 6 on "Antagonists of Medical Authority" you mention as one sort of man always found antagonistic to medical authority the Dissenting minister. I gather that the reason you assign for this attitude is that Dissenters are against authority as such.

I think here you do an injustice to Dissenters. They are obedient to God and the King; they are law-abiding citizens. But against tyranny and all powers that try to override free thought they rebel; herein surely they are the best friends of medicine and science. Ignorance and superstition are against progress in any direction; freedom and the love of freedom cannot be antagonistic to such a progressive, and at times revolutionary, science as medicine.—Yours truly,

J. N. Beadles, M.B., B.S. (Lond.).

The Hollies,
Belle Vue Road, Cinderford,
Gloucestershire,
September 9, 1913.

[It was "the Dissenting minister who follows Mr. W. T. Stead in recommending a succession of cancer-curers, faith-healers," etc., who was given, with many others, as a type of the antagonists of medical authority in the article to which Dr. Beadles refers. Dissenting ministers, as such, like their contemporaries in other denominations, cannot be criticised on this ground; they are not all followers of the late Mr. Stead. Besides, adversely to criticise a class, except in particulars, would be always foolish and ineffective.—Ed. The Hospital.]

